# The Contribution of Ribosomal Protein S1 to the Structure and Function of Qβ Replicase

**Published:** 2017

**Authors:** Z. S. Kutlubaeva, H. V. Chetverina, A. B. Chetverin

**Affiliations:** Institute of Protein Research, Institutskaya Str. 4, Pushchino, Moscow, 142290, Russia

**Keywords:** Bacteriophage Qβ, initiation, crystal structure, OB domain, ribosomal protein S1, RNA replication, RNA-directed RNA polymerase, termination

## Abstract

The high resolution crystal structure of bacterial ribosome was determined more than 10 years ago; however, it contains no information on the structure of the largest ribosomal protein, S1. This unusual protein comprises six flexibly linked domains; therefore, it lacks a fixed structure and this prevents the formation of crystals. Besides being a component of the ribosome, protein S1 also serves as one of the four subunits of Qβ replicase, the RNA-directed RNA polymerase of bacteriophage Qβ. In each case, the role of this RNA-binding protein has been thought to consist in holding the template close to the active site of the enzyme. In recent years, a breakthrough was made in studies of protein S1 within Qβ replicase. This includes the discovery of its paradoxical ability to displace RNA from the replicase complex and determining the crystal structure of its fragment capable of performing this function. The new findings call for a re-examination of the contribution of protein S1 to the structure and function of the ribosome.

## INTRODUCTION

Located on a small (30S) subparticle, protein S1 is not just the largest protein of the *Escherichia coli *ribosome, but it also has an unusual structure [1]. While other ribosomal proteins form compact globules [2, 3], protein S1 comprises a flexible strand [4, 5] almost as long as the ribosome [1] and consisting of six structurally similar units [6] called OB domains (for Oligonucleotide / oligosaccharide Binding [7]).

Protein S1 is vital to the cell, since deletions of or amber mutations in its *rps*A gene are lethal [8, 9]. However, the exact functions of protein S1 and the position of its structural domains within the ribosome remain unknown. We only know that the N-terminal segment of the protein interacts with protein S2, which is located between the head and the platform of the 30S subparticle [10]. Establishing the crystal structure of the ribosome has not helped us clarify the matter, since only ribosomes completely devoid of protein S1 have proved to be crystallizable [3]. Apparently, crystallization of the ribosomes is hindered by this prote*i*n, which lacks a fixed conformation.

Undoubtedly, protein S1, with its strong RNA-binding capacity, is important for the initiation of translation [1, 11]. However, the contribution of protein S1 to translation is not limited to the initiation step, since, unlike the initiation factors, S1 is present in the ribosome in stoichiometric amounts and remains bound to the ribosome during the elongation of a nascent polypeptide [1].

In addition to protein synthesis, protein S1 contributes to other processes that occur in the cell, both on the ribosome and outside of it [11]. One of the most known non-ribosomal functions of protein S1 is its recruitment in the synthesis of RNA as an α subunit of Qβ replicase, the RNA-dependent RNA polymerase of the bacteriophage Qβ. In addition to S1, Qβ replicase contains the phage genome-encoded catalytic β subunit and the translation elongation factors EF-Tu and EF-Ts (γ and δ subunits, respectively) [12]. The β, γ, and δ subunits constitute the Qβ replicase core, to which protein S1 is bound relatively weakly (as to the ribosome [1]) and partially dissociates during enzyme isolation [13].

Recent results of studies of protein S1 within Qβ replicase have significantly advanced our understanding of the structure and function of this protein. These results are the subject of this review.

## DOMAIN STRUCTURE OF PROTEIN S1

The “classic” OB domain consists of ≈70 amino acid residues and comprises a Greek key barrel of five β strands usually covered by an α-helix [7, 14]. OB do mains are present in the structure of many proteins capable of binding polynucleotides and polysaccharides [15]. An analysis of the amino acid sequence [6] has led to the conclusion that there are six OB domains in protein S1 (*Figure*). Subsequent structural studies have confirmed this conclusion with further refinement: the N-terminal OB domain (OB_1_) contains 4 rather than 5 β-strands [10, 16-18].

**Figure:**
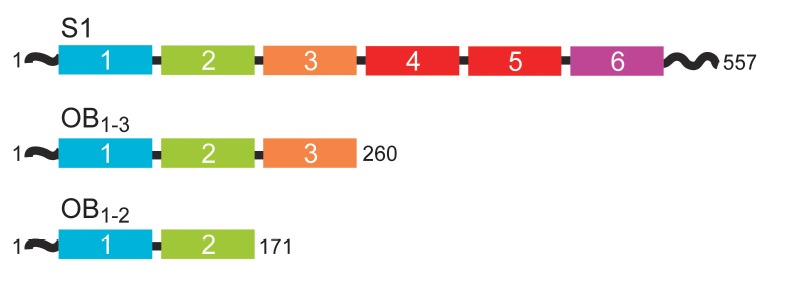
Schematic presentation of the domain structure of protein S1 and its functional fragments. OB domains are shown as numbered rectangles colored according to [16]; also indicated are the numbers of terminal amino acid residues.

The name OB domain suggests that it would display affinity for polynucleotides. Indeed, RNA-binding properties were demonstrated for domains OB3 to OB6 [1, 16, 19]. At the same time, it was thought that the domains OB1 and OB2 do not bind RNA and are involved in the protein-protein interactions responsible for the binding of protein S1 to the ribosome and to the Qβ replicase core [1]. Recent studies have demonstrated that the OB1 and OB2 domains indeed form contacts with the Qβ replicase core [17, 18], but, in addition, domain OB2 can interact with RNA due to the high density of positively charged residues on the surface area not involved in the protein-protein interaction [18]. Thus, all five classical OB domains of S1 possess RNA-binding properties.

A special role belongs to the N-terminal segment preceding domain OB1 and consisting of 20 amino acid residues. In the unbound state, this segment is unstructured [20], but upon interaction with ribosomal protein S2 [10] or the Qβ replicase core [17, 18] it forms an α helix, which is slightly longer in the latter case. Apparently, the N-terminal α-helix makes the main contribution to the interaction of protein S1 with the ribosome [19]. It is also important for the interaction of S1 with the Qβ replicase core, as suggested by both crystallographic data [17, 18] and gel filtration of protein complexes: the yield of the complex of protein S1 or its fragments with the Qβ replicase core drops sharply if this helix is removed (Z. S. Kutlubaeva, P. Seweryn and A. B. Chetverin, unpublished data).

## PROTEIN S1 AS A TERMINATION FACTOR OF RNA REPLICATION

Qβ replicase is famous for its unique ability to rapidly amplify RNA. Similar to PCR, the reaction follows an exponential kinetics, since both the original template and its complementary copy serve as templates in the next amplification round. Therefore, the number of templates increases two-fold in each round, as long as the replicase remains in molar excess over RNA. However, unlike PCR, the reaction is isothermal: there is no need to increase the temperature to melt the duplex, since the immediate reaction product comprises a single-stranded RNA. How does Qβ replicase manage to copy RNA according to the principle of complementarity, yet preserve the single strandedness of the template and the nascent strand, remains one of the unsolved mysteries of Qβ phage replication [12].

In 1972, Weissmann and colleagues published a paper [21] arguing that Qβ replicase only needs protein S1 to initiate the copying of the phage Qβ genomic (plus) RNA strand, and does not need it to copy other templates, including the Qβ RNA minus strand and the small replicating RNAs (“6S” or RQ RNAs, termed so for being Replicable by Qβ replicase). Soon after, the lead author of that paper published a review in which, by referring to unpublished results, he claimed that protein S1 is neither needed at the steps of elongation and termination of the minus strand produced by copying the Qβ RNA plus strand [22]. This view of the role of protein S1 in RNA replication persisted for the next 40 years.

Weissmann and his colleagues obtained their results when the Qβ replicase and template concentrations were similar and no exponential synthesis of RNA was possible. We found that an entirely different result was obtained when the replicase was in large excess. In that case, protein S1 dramatically stimulated the replication of both the Qβ RNA and RQ RNAs. In the presence of protein S1, most of the product was found to consist of single-stranded RNA, whereas in its absence the product was double stranded [23]. It seemed likely that protein S1 helped the replicase maintain the single strandedness of the template and the nascent RNA strand due to its known ability to bind single-stranded RNA and melt duplexes [24].

To verify this assumption, we examined the proportion between the single-stranded and double stranded forms of RNA during the elongation in the presence and absence of protein S1. As a template, we used the 4217 nt-long Qβ RNA plus strand, whose copying takes about 4 min at 30°C. To avoid an overestimation of the amount of double-stranded RNA due to its formation upon denaturation of the replicative complex [25], we tested the RNA sensitivity to ribonuclease T1 before the phenol extraction step. We found that, irrespective of the presence of protein S1, the nascent strand remained single stranded throughout the elongation step and even some time after its completion. However, while a rapid release of the single-stranded full-length product from the replicative complex was observed in the presence of protein S1, the synthesized strand remained associated with the replicative complex in its absence. Over time, a minor amount of the product spontaneously left the complex in single stranded form, whereas the major portion formed a duplex with the template and acquired resistance to ribonuclease [23].

This result showed us that protein S1 catalyzes the release of a single-stranded product from the active site of Qβ replicase: in other words, it acts as a termination factor. This function seems to be performed by protein S1 during the replication of any legitimate template [26] of Qβ replicase. As a result, both the original template and its complementary copy become available for copying in the next replication round, which provides for the exponential accumulation of RNA.

## TWO FUNCTIONS OF PROTEIN S1 HAVE DIFFERENT STRUCTURAL BASES

Thus, protein S1 performs two distinct functions during the replication of Qβ RNA: the function of a termination factor common to all legitimate templates, and a special function performed during initiation on the plus strand, previously thought to be its only function.

Our direct measurements of the rate of initiation on the plus strand of Qβ RNA showed that the requirement for protein S1 is not absolute. In a low salt buffer (50 mM NaCl), initiation in the absence of protein S1 occurred almost as rapidly as in its presence. However, the addition of 50 mM ammonium sulfate resulted in an almost complete inhibition of initiation in the absence of protein S1 and only in a two-fold inhibition in its presence (initiation on other legitimate templates was inhibited approximately two-fold regardless of the S1 presence) [23]. The addition of 100 mM of any other monovalent cation had the same effect, regardless of the nature of the anion (Z. S. Kutlubaeva, H. V. Chetverina and A. B. Chetverin, unpublished data). In view of the above, we conclude that at the initiation step protein S1 performs an anti-salt function. Apparently, when the Qβ phage RNA is replicated in *E. coli *cells, the anti-salt function is as important as the termination function because the cytoplasmic concentration of monovalent cations is even higher than 150 mM [27].

In order to determine whether all domains of protein S1 are necessary for it to perform its functions, we cloned and purified a series of N-terminal S1 fragments containing an increasing number of OB domains (*Figure*). It turned out that fragment OB_1-2_ can replace protein S1 at the termination step, while fragment OB_1-3_ can replace it in the protection of the initiation step against salt [23].

## THE STRUCTURE OF COMPLEXES OF THE Qβ-REPLICASE CORE WITH FUNCTIONAL FRAGMENTS OF PROTEIN S1

As with the ribosome, the failure of protein S1 to acquire a fixed conformation prevented the crystallization of the Qβ replicase holoenzyme. This problem was overcome when it was discovered that relatively short (and therefore less flexible) fragments can replace protein S1 in all its functions [23]. Previously, two teams (Danish-Russian and Japanese) had solved independently of each other the crystal structure of the Qβ replicase core [28, 29]. Recently, the same teams independently solved the structure of a complex containing the core and the first two OB domains of protein S1 [17, 18, 30]. Although the Japanese group investigated the crystals of the replicase core complexed with fragment OB_1-3_ [17] while the Danish-Russian team studied the complex with fragment OB_1-2_ [18], the same structural information was obtained in each case, since the third OB domain was not visible, due to the fact that it was unfixed within the structure of the complex [17]. In addition to contributing to our understanding of the mechanism of RNA replication, these studies are interesting in that they have established the crystal structure of a 1/3 of the protein S1 molecule, precisely the part whose structure was the least studied.

The binding of domains OB_1_ and OB_2_ produces almost no effect on the structure of the Qβ replicase core. These domains interact with the β-subunit in the region of the “fingers” domain, which participates in the formation of the active site of replicase, the binding of RNA, and the unwinding of the complementary strands of the replicative complex [28, 31]. The N-terminal α-helix of protein S1 is located between domains OB_1_ and OB_2_ and forms a number of contacts with the β-subunit and EF-Tu [18]. These contacts are similar to those the helix forms with the ribosomal protein S2 [10].

Although the two research groups reported nearly identical structural data [17, 18], they drew somewhat different conclusions. Thus, the Japanese group claimed that domains OB_1_ and OB_2_ do not have basic and aromatic amino acid residues capable of forming bonds with the phosphates and nitrogenous bases of RNA, and therefore cannot interact with RNA [17]. On the contrary, the other group discovered an extended, positively charged region on the surface of domain OB_2_ and presented NMR data demonstrating the ability of this domain to bind RNA [18].

## THE CONTRIBUTION OF PROTEIN S1 TO THE INITIATION OF RNA REPLICATION

This function is similar to the one commonly assigned to protein S1 when considering its role in translation. The difference is that, while protein S1 stimulates initiation of the translation of the vast majority of mRNAs [1, 11], it promotes the initiation of replication of only one of a variety of Qβ replicase templates. Unlike other templates, the initiation on the Qβ RNA plus strand requires that the replicase binds the RNA not only at the 3’ end wherein it begins copying, but also at the “M site,” an internal template site spaced by ≈1,500 nt from its 3’ end. In the absence of protein S1, the replicase cannot bind the M site [32]. Apparently, protein S1 stimulates the initiation on the Qβ RNA plus strand by increasing the replicase affinity to the M site. This is supported by the fact that initiation becomes sensitive to the elevated salt concentration both in the absence of S1 protein [23] and in its presence, if certain mutations are introduced into the M site [33]. The ability of fragment OB_1-3_, rather than OB_1-2_, to replace protein S1 in the anti-salt function means that the third OB domain plays a major role in the interaction with the M site, whereas domains OB_1_ and OB_2_ are needed as far as they form a link between domain OB_3_ and the replicase core.

## THE CONTRIBUTION OF S1 PROTEIN TO THE TERMINATION OF RNA REPLICATION

The Japanese group proposed a mechanism for protein S1 action in which the mobile domain OB_3_ plays a key role in both the initiation and termination steps, by interacting with the M site of the Qβ RNA plus strand during initiation, and with the newly synthesized RNA strand during termination [17, 34]. Although the authors referred to our paper [23], they apparently read it inattentively, since the paper directly showed that during termination protein S1 was replaced by its fragment OB_1-2_, in which domain OB_3_ was absent and, therefore, could not participate. Incidentally, neither did they notice the fact that the same paper demonstrated the key role of domain OB_3_ at the initiation step one and a half year prior to their publication.

The Danish-Russian paper reports that the positively charged region of domain OB_2_ adjoins a similarly charged region on the surface of the β subunit and forms a continuous, positively charged tract leading from the opening through which the synthesized strand is thought to be released from the active site [18]. Probably, this tract is essential for the release of the synthesized strand from the replicative complex.

However, it would be premature to propose a detailed mechanism of termination, since the product is terminated from the closed conformation of Qβ replicase [26], whereas the reported structure of the core : fragment OB_1-2_ complex represents the open conformation. In this regard, we would note that protein S1 catalyzes the termination step even if it is added during the elongation step, but before its completion [23]. In other words, there is a “no return point” somewhere at the end of the elongation after which protein S1 cannot promote the release of the synthesized strand. What is that point?

As a result of the initiation on a legitimate template in the presence of GTP, Qβ replicase acquires a closed conformation from which neither the template nor its complementary copy can dissociate until the elongation is complete [26]. This ensures high processivity of the replicase, but it hinders the evacuation of its active site after the copy is completed. To ensure “recycling” of the enzyme, the closed conformation must be converted back to the open one. Probably, it is this transition that is induced by the mysterious untemplated 3’-terminal adenylylation of the synthesized strand, which precedes its termination [12] and represents the very moment by which protein S1 has to be embedded into the replicase molecule in order to fulfill the function of a termination factor.

In conclusion, we would like to note that the discovery of the ability of protein S1 to displace RNA from a complex changes the basic paradigm according to which the only purpose of this protein is to hold RNA near the active site of an enzyme, whether it is a replicase or a ribosome [1], and calls for a re-evaluation of the possible role of S1 in translation and other cellular processes.
